# Phagocytosis–Inflammation Crosstalk in Sepsis: New Avenues for Therapeutic Intervention

**DOI:** 10.1097/SHK.0000000000001541

**Published:** 2020-06-08

**Authors:** Marcela Hortová-Kohoutková, Federico Tidu, Marco De Zuani, Vladimír Šrámek, Martin Helán, Jan Frič

**Affiliations:** ∗ International Clinical Research Center, St. Anne's University Hospital Brno, Brno, Czech Republic; † Department of Anesthesiology and Intensive Care, Faculty of Medicine, Masaryk University, Brno, Czech Republic; ‡ Institute of Hematology and Blood Transfusion, Prague, Czech Republic

**Keywords:** Monocyte, phagocytosis, sepsis, signaling

## Abstract

Phagocytosis is a complex process by which cells within most organ systems remove pathogens and cell debris. Phagocytosis is usually followed by inflammatory pathway activation, which promotes pathogen elimination and inhibits pathogen growth. Delayed pathogen elimination is the first step in sepsis development and a key factor in sepsis resolution. Phagocytosis thus has an important role during sepsis and likely contributes to all of its clinical stages. However, only a few studies have specifically explored and characterized phagocytic activity during sepsis. Here, we describe the phagocytic processes that occur as part of the immune response preceding sepsis onset and identify the elements of phagocytosis that might constitute a predictive marker of sepsis outcomes. First, we detail the key features of phagocytosis, including the main receptors and signaling hallmarks associated with different phagocytic processes. We then discuss how the initial events of phagosome formation and cytoskeletal remodeling might be associated with known sepsis features, such as a cytokine-driven hyperinflammatory response and immunosuppression. Finally, we highlight the unresolved mechanisms of sepsis development and progression and the need for cross-disciplinary approaches to link the clinical complexity of the disease with basic cellular and molecular mechanisms.

## INTRODUCTION

**Fig. 1:**
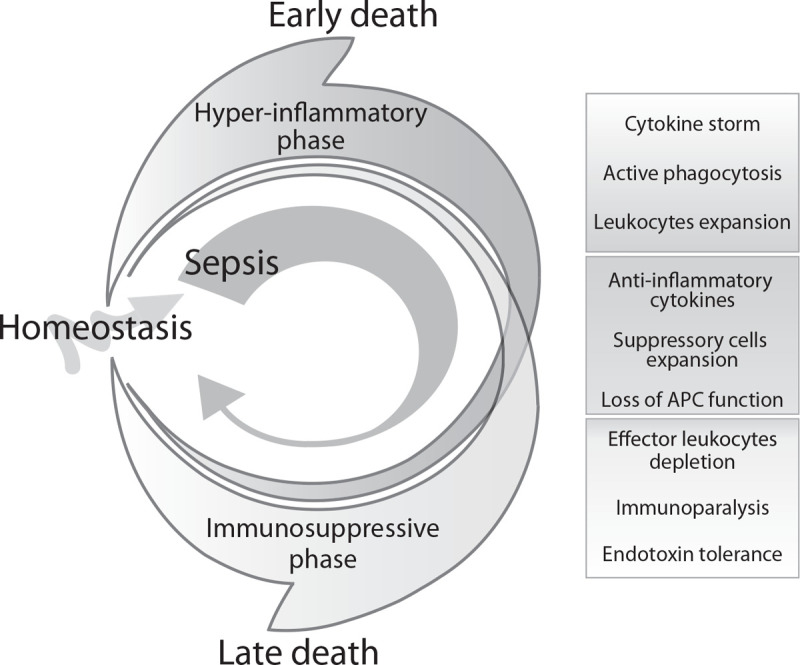
Dynamic changes in the immune system during sepsis progression.

Sepsis is a serious life-threatening condition that affects ∼49 million people and contributes to 11 million deaths worldwide every year (1). Patients with sepsis experience an excessive systemic inflammatory reaction to microbial infection, perhaps due to insufficient phagocytosis and cellular clearnace of pathogen. Sepsis is characterized by an initial hyperinflammatory phase and a later immunosuppressive phase (2, 3). During the initial phase, a broad spectrum of cytokines, chemotactic mediators, and other effector molecules is secreted which influences phagocytic events and the downstream signaling processes. The later immunosuppressive phase is characterized by reduced activity of phagocytes, metabolic rewiring of monocytes, reduced functionality of antigen presenting cells associated with decreased MHC-II expression, expanded myeloid-derived suppressor cells, and depleted effector cells (3, 4) (Fig. 1).

During initial phase of sepsis, the hyperinflammatory phase develops and is associated with cytokine storm, active removing of pathogens by phagocytosis and expansion of different leukocyte types. Too excessive hyperinflammatory phase can lead to early death of septic patients. During progression of sepsis, production of anti-inflammatory cytokines is progressively augmented leading to disbalance of pro- and anti-inflammatory cytokines levels. This fact also contributes to the expansion of suppressory cells and loss of antigen presenting function. Subsequently, when the immunosuppressive phase completely prevails, the depletion of effectory cells contributes to immunoparalysis of immune system. The disproportionate levels during immunosuppressive phase can cause late death of patients during progression of sepsis. The immunoparalysis as well as endotoxin tolerance can persis also when the homeostasis is restored.

Current sepsis treatment includes initial antibiotic administration but can further escalate to organ supporting therapies (such as artificial ventilation, circulatory support, and renal replacement therapy) if the patient fails to respond. Therapies that specifically target pathophysiological mechanisms are lacking but urgently needed, as the number of sepsis cases are likely to increase due to the growing proportion of antibiotic resistant pathogens, immune-compromised individuals, and the aged population (5, 6). One such pathophysiological mechanism underlying sepsis might be phagocytosis.

Phagocytosis is a crucial cellular mechanism devoted to eliminating pathogens and damaged or infected cells, promoting tissue regeneration, and ensuring homeostasis. Through this process, particles >0.5 μm are recognized via phagocyte transmembrane surface receptors and ingested into membrane-derived vesicles, known as phagosomes. These phagosomes later fuse with lysosomes for complete cargo digestion (7, 8). Here, we focus on the effects that sepsis and its pathophysiology might have on phagocytosis and vice versa. First, we outline the major events of the phagocytic process. Then, we highlight how intense cytokine expression and release during phagocytosis contributes to the “cytokine storm” that occurs in the initial phase of sepsis. We also identify how the molecular control of phagocytosis in the latter sepsis phase might affect the pathology outcome. Furthermore, we link some of the events in phagocytosis–inflammation crosstalk with therapeutic interventions currently used to control sepsis. We conclude by proposing new connections among phagocytic processes, sepsis mechanisms, and treatment solutions.

## RECOGNITION RECEPTORS AND PHAGOCYTOSIS CONTROL IN SEPSIS

**Fig. 2:**
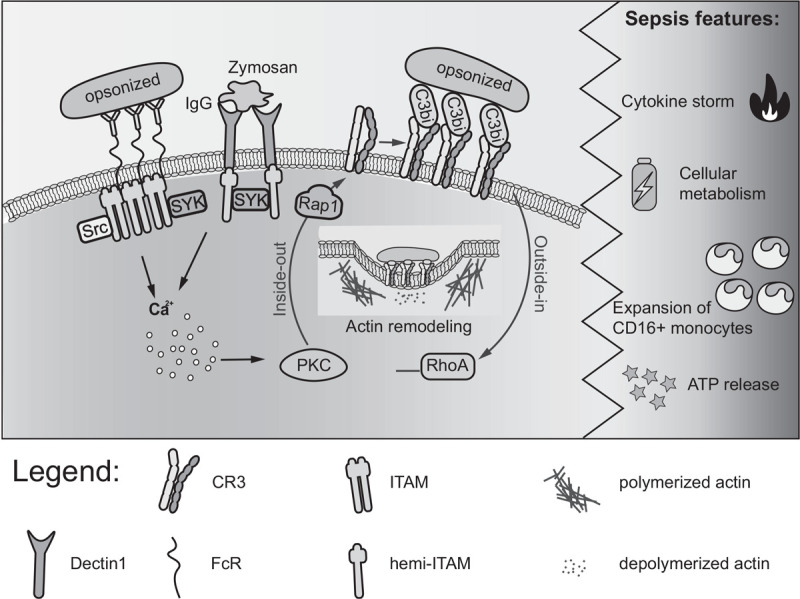
Phagocytosis: the main receptors and down-stream signaling outcomes.

Phagocytosis progresses through four main steps: activation; chemotaxis; attachment; and ingestion (9). These steps revolve around the energetically demanding process of actin filament polymerization and depolymerization that facilitates phagocytic receptor mobility, pathogen detection, and engulfment (10). These four steps rely on a tightly tuned signaling network driven by various phagocytic receptors (9, 11–13) categorized as opsonic, nonopsonic, and integrins (Table 1 and Fig. 2). Opsonic receptors, mainly Fc receptors (FcR), bind antibodies coating the pathogen surface (14). Nonopsonic receptors directly recognize the pathogen-associated molecular patterns (PAMPs) or acts as scavenger receptors such as MARCO, CD36, SR-BI/II (15–18). Finally, the integrins have an important role in activating immune pathways and initiating cytoskeletal rearrangements (19). Orchestrated receptor signaling in sepsis is thus crucial for the timely detection and elimination of localized pathogens and the subsequent prevention of a systemic infection and endotoxin circulation.

**Table 1:**
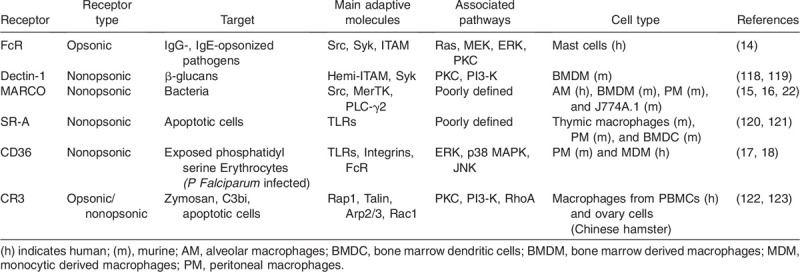
Phagocytic receptors that recognize opsonized and nonopsonized targets

Recognition of IgG-coated particles leads to FcR clustering on the cell membrane, followed by Src kinase activation and phosphorylation of the ITAM motif present in the cytoplasmic domain. Once phosphorylated, ITAMs expose a docking site for Syk (124). The Src–ITAM–Syk complex triggers calcium signaling and eventually guides phagosome formation and invagination (125). Dectin-1 is a C-type lectin receptor: particulate β-glucan recognition leads to Dectin-1 clustering in the phagocytic area. The recognition of the target initiates signal transduction through an ITAM-like domain, which is phosphorylated by Src kinases and allows the recruitment of Syk kinases to drive downstream signaling (126). CR3 recognizes targets opsonized by C3bi (127). In this case, “inside-out” signaling increases the affinity of CR3 for its ligand C3bi, an opsonin coating the surface of phagocytic preys. The binding of this C3bi-opsonized particle to the integrin receptor CR3 triggers “outside-in” signaling, which is responsible for Rho-dependent actin polymerization (128). During the sepsis, the overall outcomes of phagocytosis are influenced by systemic consequences of the inflammatory process (right panel).

The recognition of soluble versus particulate pathogens is important for activating the appropriate inflammatory signaling pathways (20, 21). Indeed, in the context of sepsis, several pathogen evasion strategies have been reported which are based on blocking phagocytic processes (22–24). Despite these clear links between phagocytosis and sepsis risk, only a few studies have focused on the impact of phagocytosis during sepsis progression to date. In a typical sepsis mouse model based on cecal ligation and puncture (CLP) (25), Liu et al. (26) showed that knock-out of CD11b, a component of the CR3 integrin receptor, was associated with poorer outcomes, higher bacterial load, systemic inflammation, and splenic apoptosis compared with wild-type control mice. Consistently, impaired integrin signaling and in particular CR3, potentiates bacterial sepsis in mice (26, 27). However, the use of a monoclonal antibody capable of blocking the interaction between CR3 and its pro-inflammatory ligand CD40L elicited only a mild increase in the phagocytic capabilities of isolated murine macrophages (28). Leelahavanichkul et al. (29) improved the sepsis response in a mouse model by blocking phagocytic scavenger receptors. Specifically, CLP knock-out mice for either CD36 or CR-BI/II, two nonopsonic receptors belonging to the family of class B scavenger receptors (SR-B), exhibited increased survival, limited bacterial proliferation, and reduced systemic inflammation compared with control mice (29). The authors suggested that while SR-B receptors are responsible for both pathogen-induced signaling and pathogen-triggered phagocytosis, under conditions of incomplete phagocytosis or lysosomal bacterial escape, they can also contribute to intracellular bacterial growth. These conclusions remain controversial as opposing findings were reported by Guo et al. (30). Interestingly, de Tymowski et al. recently showed that the IgA FcR CD89 can directly bind to bacteria even in the absence of its cognate ligands (IgA and CRP). Furthermore, CD89 transgenic mice were protected against CLP and preumonia-induced polymicrobial sepsis, highlighting the essential role of the FcR during the early phase of infection before specific antibodies are produced (31).

These examples pinpoint the need to investigate phagocytic receptors in the context of sepsis. Targeting such receptors has thus far led to promising results in murine models but validation in clinical studies is still lacking. Nonetheless, the nature of the receptors, their parallel roles in eliciting detrimental high-grade inflammation, and their efficiency in recognizing and clearing bacteria seem to be important parameters that should be carefully addressed in a therapeutic context.

## SEPSIS-ASSOCIATED CHANGES TO PHAGOCYTE SUBSETS

### Phagocytic activity

The different phagocytes share receptor profiles, pathogen engulfing ability, and also some intracellular processes (such as phagolysosome formation or respiratory burst) maintain subset-specific roles (32, 33). During sepsis, the presence of pathogens and high levels of inflammatory cytokines results in a dynamic change in the proportion of leukocytes in the blood (34). Neutrophils are the most common phagocytic cells in the blood, constituting 60% to 80% of blood leukocyte counts; their production and release from the bone marrow can increase up to 10-fold upon infection (35, 36). Studies using CLP models, and observations of humans suffering from sepsis, have shown that the bacterial endotoxins and pro-inflammatory cytokines released in response to infection induce substantial rigidity to the neutrophil membrane and reduce neutrophil adhesion, margination, and rolling along the blood vessel endothelium, resulting in impaired migration to the inflammed tissue (37, 38). Interestingly, this phenomenon seems to only occur during lethal sepsis and is linked to an early downregulation of CD18 (a key component of ICAM-1-binding integrins) on the polymorphonuclear cell (PMN) surface (39). Consequently, increased neutrophil membrane rigidity is associated with sepsis severity (38), although the molecular mechanism remains unknown. Others have shown that the phagocytic activity of neutrophils and monocytes has a direct influence on severe sepsis outcomes (40). Specifically, Danikas et al. (40) showed that a worse prognosis and survival in affected patients correlated with decreased expression of CD64 (a Fc receptor associated with neutrophil activation and phagocytosis of antibody-opsonized particles) and with impaired neutrophil phagocytic activity. Based on these results, the researchers proposed that PMN phagocytic activity might serve as a prognostic indicator for sepsis. As the patients were uniformly recruited 24 h after ICU admission, it remains possible that the differences observed in phagocytic activity might be a consequence of a different phase of pathology progression. Another study performed on a septic cohort reported sepsis-associated expansion of circulating immature neutrophils with low phagocytic activity (41).

### CD16^+^ monocyte expansion

Human monocytes represent 2% to 8% of blood leukocyte counts and can be categorized into three major subpopulations: classical (CD14^++^CD16^−^), intermediate (CD14^++^CD16^+^), and patrolling (CD14^+^CD16^++^) monocytes (42). Sepsis leads to the expansion of CD14^++^CD16^+^ monocytes (43–45) but the clinical relevance of this expansion is unclear. Others have observed that an increased level of intermediate and patrolling monocytes in the peripheral blood correlates with a better sepsis prognosis (46, 47). In healthy individuals, classical monocytes are highly phagocytic compared with the other two subsets (44, 48, 49). Differences in phagocytic ability between monocyte populations are also observed in sepsis (45). A recent study showed an expansion of CD16^++^ patrolling monocytes in early phase septic patients followed by gradual reduction in 7 and 14 days after ICU admission (50). During the late immunosuppressive phase of sepsis, the phagocytic activity of monocytes and pro-inflammatory cytokine expression is notably reduced in septic patients (51), undoubtedly contributing to functional decline and immune paralysis.

### Monocyte endotoxin tolerance

Monocytes that are acutely exposed to endotoxin develop a transient unresponsive state toward subsequent LPS challenges, a paradigm known as endotoxin tolerance (ET) (52). Although the underlying mechanisms of ET are debated, this phenotype seems to be characterized by altered cytokine release and monocyte effector functions due to complex rewiring of metabolic pathways (which, in turn, control specific epigenetic signatures), molecular signaling pathways, and transcription factor activation (52–56). Although ET monocytes produce less pro-inflammatory cytokines (as TNF-α and IL-6), they increase their phagocytic activity, as evidenced by the upregulation of genes involved in phagocytosis (e.g., Fc receptors CD64 and CD23, MARCO), reactive oxygen species (ROS) production and wound-healing capacity, while showing impaired antigen presentation (57–59). Even though the classic dichotomy of M1/M2 macrophage subsets does not reflect the complexity of tissue macrophages, especially in clinical setups, this phenotype closely resembles the one of M2-like macrophages (57).

Interestingly, in a model of experimental peritonitis in baboons, a more pronounced expansion of M2-like circulating monocytes was found in surviving animals compared with non-survivors. Interestingly, the non-survivors showed mostly M1-like monocyte expansion (60). A recent study using a human *in vivo* model of endotoxemia, however, showed that ET monocytes cannot induce an oxidative response to *Escherichia coli* or *Candida albicans* challenge. This effect was due to a failure of ET monocytes to modify their metabolism during *ex vivo* LPS restimulation, resulting in reduced *Candida* killing capacity (54). However, as most of these studies used different protocols to induce ET, both *in vivo* and *ex vivo*, the unambiguous interpretation of the process remains challenging.

Monocytes isolated from patients with sepsis exhibit several features reminiscent of ET monocytes, including reduced cytokine production after LPS exposure and increased phagocytic ability (61–63). Two recent studies performed on the same cohort demonstrated that circulating monocytes also show a shift toward an M2-like phenotype, where the levels of CD86, HLA-DR, PD-1, and PD-L1 positively correlated with patient survival (50). These same monocytes showed no difference in phagocytosis ability compared with healthy volunteers, but increased production of ROS and nitric oxide (NO) (63). Consistent with the first study, high CD64 levels in patients during sepsis correlated with increased PMN phagocytosis and better clinical outcomes, while HLA-DR down-regulation correlated with worse clinical outcomes (40, 64). Another study on a septic cohort showed that neutrophil and monocyte functions, including those related to phagocytosis, progressively diminished as sepsis persisted (65). This phenomenon correlated with increased PD-L1 expression on neutrophils and monocytes; consequently, the condition was reversed upon incubating these cells with PD-1 or PD-L1 blocking antibodies, which helped to restore phagocyte function (65).

Taken together, these data suggest that phagocytosis has a key role during sepsis resolution and the mechanisms underlying phenotypic shifts in monocytes and neutrophils might be crucial for designing novel therapeutic approaches. The limitations arising from simplicistic phenotypeing of myeloid subsets (e.g., M1/M2) must now be overcome using new techniques such as single-cell sequencing of tissue macrophages or circulating monocytes, to provide detailed pathology-specific results. Despite the remaining controversies among clinical studies, a further detailed assessment of phagocytic process might help identify novel markers of sepsis severity.

## CYTOKINE-MEDIATED REGULATION OF PHAGOCYTOSIS IN SEPSIS

As discussed, microbe internalization by phagocytes is accompanied by an inflammatory cascade and cytokine secretion. These processes are mediated mainly via pattern recognition receptors (PRRs), thus constituting a functional link between phagocytosis and PRR-driven inflammation (21). Together with phagocytic efficacy, cytokine expression levels are among the most significantly divergent parameters between patients with sepsis, and thus can be used to guide patient stratification (66).

The acute phase of sepsis is associated with a deregulated auto-amplifying secretion of pro-inflammatory cytokines, which transmits the “danger signal” of invading pathogens and alerts the host defense. Pro-inflammatory cytokines are secreted in high levels during the hyperinflammatory phase of sepsis, suggesting that cytokines might be able to modulate phagocytosis itself. Indeed, IL-1β, IL-8, TNF-α, and IFN-γ can enhance the phagocytic function of human neutrophils but they are not involved in the bactericidal functions of these cells (67). A similar effect was also shown in a murine macrophage cell line, where IL-18 boosted phagocytosis efficacy (68). IL-6, however, improves the bactericidal effects of neutrophils without any impact on phagocytosis (67).

High IL-10 levels serve as a marker of the immunosuppressive phase of sepsis associated with a poor patient prognosis (69). Non-surviving patients affected by severe sepsis exhibit high IL-10 serum levels and low HLA-DR expression on circulating monocytes compared with surviving patients (64). Conversely, surviving patients exhibit higher TGF-β1 serum levels than non-survival patients; however, the underlying mechanism of this effect is unclear (64). Interestingly, IL-10 can interfere with phagocytosis by blocking phagosome maturation (70) and inhibiting the expression of MHC II and other costimulatory molecules in monocytes and macrophages (71). This mechanism might, therefore, underlie the differential patient outcomes reported based on IL-10 expression levels.

Given the growing body of evidence on the role of cytokines in sepsis, many researchers have attempted to develop specific anti-cytokine-based therapies (72–74). Efforts have also been made to develop and test various blood purification methods to remove endotoxins and/or cytokines from patients, providing partially promising results, such as early circulatory stabilization and prevention of organ failure (74). However, patients affected by sepsis can also profit from some cytokine-based therapies. GM-CSF, a myelopoietic growth factor, has been proposed for the treatment of sepsis (75, 76) as GM-CSF is a strong activator of phagocytosis and promotes phagocyte infiltration and expansion in inflamed tissues (77).

Interestingly, the cytokine storm typically observed during sepsis is not exclusively induced by microbial infection. Many other life-threatening, noninfectious causes such as shock states, trauma or ischemia–reperfusion injury are associated with systemic inflammatory response syndrome (SIRS) (78). Although the etiology of these pathologies is different, human SIRS and sepsis share similar patterns of cytokine expression (79). Like in sepsis, this robust inflammatory storm is also associated with the extensive formation of cell debris arising from apoptosis (80) and necroptosis (81) of many different cell types, which needs to be cleared by phagocytes. During these processes, several damage-associated molecular patterns (DAMPs) (such as S100A8 and S100A9) are released into the bloodstream. These DAMPs act as endogenous TLR4 ligands and are able to induce a state of “tolerance” in monocytes, similar to that observed after LPS challenge (82). Moreover, SIRS can induce profound changes in gastro-intersticial (GI) permeability (83, 84), which leads to the translocation of symbiotic bacteria from the GI tract to the blood stream, finally resulting in multiple amplification of inflammatory responses and switching “sterile” SIRS into “nonsterile” with bacterial (PAMPs) immune-stimulation.

## IMBALANCED ENERGY METABOLISM AND PHAGOCYTOSIS: A POSSIBLE CROSSTALK IN SEPSIS?

A key characteristic of sepsis is imbalanced cellular energy metabolism (85, 86), which contributes to immune paralysis and vulnerability to secondary infections (87). Although the involvement of ROS and NO during sepsis is debated, as boosting and inhibiting their activity has opposing effects (88, 89), they also have an indirect role in phagocyte metabolic switch during the sepsis (90). The initial phase of sepsis is characterized by elevated mitochondrial respiration and ATP production (91). When the overproduction of inflammatory molecules such as ROS and NO disrupts mitochondrial respiration by the oxidation of respiratory enzymes, the main source for ATP generation is substituted by enhanced glycolysis (92, 93). The possible effects of the energy source on phagocytosis can be demonstrated on M1-like and M2-like macrophages: while M2-like macrophages produce ATP through mitochondrial respiration and show a high level of phagocytosis, the less phagocytic M1-like macrophages mostly rely on glycolysis (94). M1-like and M2-like macrophages also differ in phagosome metabolic activity. Whereas M1-like macrophages rely mostly on the generation of superoxides to eliminate pathogens and acidify their phagosome quite slowly, M2-like macrophages promptly ensure phagosome acidification to quickly and effectively clear apoptotic cells (95). Nevertheless, M2-like macrophages are able to produce IL-10, which can inhibit phagosome maturation (70). Finally, because M2-like macrophages are mostly involved in clearing apoptotic cells rather then bacteria and can release immunosuppressive chemokines, it is likely that these cells participate in the immunosuppressive phase of the pathology (50, 63, 96, 97).

Damaged cells and tissues release large quantities of ATP into the extracellular space. This ATP acts as a DAMP, signaling through PRRs as a paracrine and autocrine modulator of cellular responses (98). Zumerle et al. (99) demonstrated that ATP released by immune cells rapidly transmits danger signals between cells and boosts phagocytosis through purinergic signaling and calcium release. Consistently, blocking the purinergic receptors P2X4 and P2X7 on macrophages reduces their phagocytic activity (99).

To sustain the high-energy demands during acute catabolic responses in sepsis, proper therapeutic nutrition support is generally needed. Interestingly, increased catabolism can persist for up to 2 years after sepsis (100). The high demand of essential nutrients is also highlighted by the obesity paradox: obese and severely obese individuals have a better prognosis during the first year after sepsis compared with nonobese patients (101).

We believe that such severe metabolic defects reported in septic patients might be considered as new therapeutic targets. For example, IFNγ administration to septic patients can restore energy metabolism and reverse immunoparalysis (85). Consistent with the impairment of mTOR pathways described in monocytes isolated from septic patients, inhibiting mTOR-dependent metabolic processes with metformin in a murine model of fungal sepsis resulted in decreased cytokine production and higher mortality (85). We speculate that the decrease of phagocytic activity in late phase of sepsis might thus be linked to dynamic changes in phagocytes’ energy metabolism.

## ROUTINE THERAPEUTIC APPROACHES INFLUENCING PHAGOCYTIC PROCESSES

Our knowledge of the molecular control of phagocytosis has, to date, had little impact on therapeutic approaches in sepsis. As mentioned above, specific phagocytosis-targeted therapy is not in routine use but some routine approaches influence phagocytosis as an off-target effect. Phagocytosis is affected by many physico-chemical factors, many of which are readily influenced in critically ill patients. For example, an elevation in phagocytic capacity has been reported in conditions such as hyperthermia (102), hypoxia (103), and high insulin levels (104). All of these conditions can be a component of early sepsis syndrome and we suggest that they might be part of an evolutionary adaptation to systemic infection. Conversely, diminished phagocytosis has been reported in hypercapnia (105), hypothermia and hyperoxia (106, 107). As a result, organ-supporting strategies in sepsis treatment could be potentially harmful, at least in the context of phagocytosis. For example, ventilator support and efforts to increase oxygen delivery to hypoxic peripheral tissues during septic shock may lead to long-lasting hyperoxia in other tissues, especially in the lungs. The phagocytic activity of pulmonary macrophages is impaired in hyperoxic conditions (106) and long-term hyperoxia is associated with a high incidence of complicating pneumonia (107).

Similarly, some routinely used drugs may affect the activity of immune cells or their phagocytic capabilities. Antibiotics, which a crucial in sepsis therapy, have been intensively studied for their antibacterial effects but they also have rich immunomodulatory potential (108). The beneficial immunomodulatory effects of macrolides have been intensively discussed (108); these have been shown to reduce the mortality of community-acquired pneumonia when used as a part of combination therapy (109). Clindamycin, erythromycin, and chloramphenicol have all been described to increase phagocyte functions, whereas tetracycline, gentamicin, and ciprofloxacin seem to have the opposite effect (110, 111). Catecholamines, epinephrine, and norepinephrine promote M2-like macrophage polarization and enhance macrophage phagocytotic activity via β2-adrenergic receptors *in vitro*(112, 113). Similarly, another routinely used vasoactive drug, vasopressin, can enhance monocyte and neutrophil functions — namely migration and chemotaxis (114).

Although recommended by current Surviving Sepsis Campaign guidelines (115), the safety and efficacy of corticosteroids in septic shock is also controversial. The primary goal of corticoid therapy is to substitute for sepsis-induced adrenal insufficiency, but clearly it may also have an immunomodulatory effect. In the context of phagocytosis, preliminary data suggest that corticoid treatment might amplify neutrophil-mediated phagocytosis in the early phases of septic shock (116, 117).

## CONCLUDING REMARKS

Most of the key events in sepsis, including the dynamic changes in metabolism, cytokine expression, and immune signaling, are associated with the initiation and control of phagocytic processes. These findings imply that intervening on monocytes and neutrophils to enhance phagocytosis during the course of sepsis (both in the early and late phases) could have clinical benefits. To date, most therapeutic approaches tackling sepsis do impact on phagocytic processes. Nevertheless, the detailed mechanistic link between the therapy and phagocytosis is elusive, meaning that this key point of potential intervention thus far has been overlooked. We believe that a specific approach to enhance and prolong the phagocytic capacity of cells would promote sepsis resolution. Unfortunately, most of the data linking sepsis pathobiology and phagocytosis have been generated indirectly or reported in isolated studies, frequently addressing sepsis progression markers than the potential treatment strategies. Going forward, future research should aim to address phagocytosis directly in larger clinical studies, this might suggest new therapeutic targets enhancing the phagocytic process and clearance of pathogens.
